# Annotations

**Published:** 1904-01-09

**Authors:** 


### Annotations.

### The Study of Children's Diseases.

It must be admitted that the study of disease in children has not been pursued in this country with anything like the vigour and success that have marked the enterprise of physicians in America and certain continental countries. Whatever be the explanation, the fact is beyond question. There have been, it is true, a few distinguished exceptions, but whilst affording to these due recognition it must be allowed that the general body of physicians and surgeons have treated this department of practice in a somewhat apathetic manner. Hence advances in doctrine and practice have come mainly from abroad rather than from Great Britain. There are, however, signs that John Bull is waking up. The first of these was the establishment of a special society for the study of disease in children. This has now been in existence for three years, and its influence is beginning to be felt. Its annual volume of "Transactions" presents a goodly record of work, and we are glad to notice contributions from gentlemen in general practice as it is obvious that those who have the advantage of such an experience enjoy particularly good opportunities in this direction. Another praiseworthy feature of the society is the arrangement according to which one meeting in each session is held in a provincial town. In some of these work in this department is decidedly in advance of the Metropolis. The most recent evidence of an increased interest in the study of disease in children is the publication of a new journal entitled the *British Journal of Children's Diseases* under the editorial guidance of Dr. George Carpenter. The first number has just been issued and contains several important papers. Considering the extensive clinical opportunities of our great cities, there is no reason why this country should not take a leading position in work of this order. One essential, or at least important condition is increased recognition of the importance of the subject in our medical schools. These, as has often been the case heretofore, must be aroused to their duty by pressure applied from the outside.

### The Mode of Infection by Plague.

In the official report, issued by the Board of Health of New South Wales, on the second outbreak of plague at Sydney, Dr. J. Ashburton Thompson, Chief Medical Officer of the Government, makes some interesting observations relative to the possible convection of plague virus by rat fleas. The main conclusion at which he arrives is that observation of the field-facts, as he calls them, appears to substantiate the inference, already gained from laboratory experiments, that plague is communicated to man by fleas which have previously fed upon infected rats. Over and over again the establishment of new centres of infection was noticed at distant and widely separated points to which fodder had been transported from a line of wharves already known to be infested with plague rats; and precedence of the disease in these animals over its occurrence in man was remarked at those localised centres. Another peculiarity was frequently observed, namely, that among a number of individuals working in a warehouse infested with plague-stricken rats, perhaps only one man was attacked by plague, although there seemed little doubt that the rest were equally exposed to infection. These facts, together with many others, led Dr. Thompson to incline towards the theory that rat-fleas are the main agents in transmitting the disease to man. An inquiry was made in order to discover what species of fleas may be found on rats, and four different species were found. The kind occurring most abundantly was pulex pallidus, and in three instances plague bacilli were found in members of this species obtained from plague-infected rats. The outcome of the inquiry is to indicate, according to Dr. Thompson, that the promise of safety in the future lies neither with attempts to prevent the importation of plague rats, nor with attempts to exterminate rats infesting the locality to be defended, but in habitually excluding these vermin from inhabited premises.

### Rainfall and Public Health.

The excessive rainfall of the past year has not hitherto been apparently injurious to the public health as measured by the death-rates, which have been below the average. Indeed this comparative freedom from disease is what might have been expected, for the saturated state of the soil is not really its most dangerous state for the public health. On the endemic soils of cholera, plague, and malarial fever, the swamping of the ground not only does not increase the number of cases, but may nearly extinguish the disease for a few weeks. The ill effects are felt only when the level of the water in the ground begins to sink, getting worse in cholera and plague until drought is reached. What is observed on the great scale and with a good deal of uniformity in these foreign diseases, has been seen also at home in the seasonal maxima of enteric fever, as well as of cholera when it has been epidemic, and of plague in former times. The first two seasons of the nineteenth century, when enteric was brought prominently to notice, the summers and autumns of 1826 and 1846, were both exceptionally hot and dry. It is the extreme fluctuations from a flooded to a parched state of the ground that have often made the danger to health; and where these recur with the regularity of the seasons themselves, as in Bengal or Bombay, the risks of the hot and dry season in one form or another are perennial. With us the degrees of saturation and drought keep nearer a mean, and even when the fluctuation is abnormal it is never so extreme as in the countries of the monsoon. But there is no doubt that, in our latitudes also, a fall of the water in the soil from a very high level is often apt to be attended by an increase of certain "zymotic" diseases. Any effects of the present wet season in that way will depend largely upon the character of the season following. It is not easy to explain this empirical law; but, according to the well-known teaching of the Munich school, it is that the decomposition processes in the porous soil become of a noxious kind when the pores of the ground are partially or wholly occupied by air after having been completely filled by water.

